# Supplementary material for: Slow kinesin-dependent microtubular transport facilitates ribbon synapse assembly in developing cochlear inner hair cells
Source: bioRxiv. 2024 Apr 15:2024.04.12.589153. Preprint. [Version 1] doi: 10.1101/2024.04.12.589153 (PMC11042220; doi:10.1101/2024.04.12.589153)
Supplement: Supplement 1 [file NIHPP2024.04.12.589153v1-supplement-1.pdf]

bioRxiv preprint doi: https://doi.org/10.1101/2024.04.12.589153; this version posted April 16, 2024. The copyright holder for this preprint (which was not certified by peer review) is the author/funder, who has granted bioRxiv a license to display the preprint in perpetuity. It is made available under aCC-BY 4.0 International license.

Supplemental Figures:

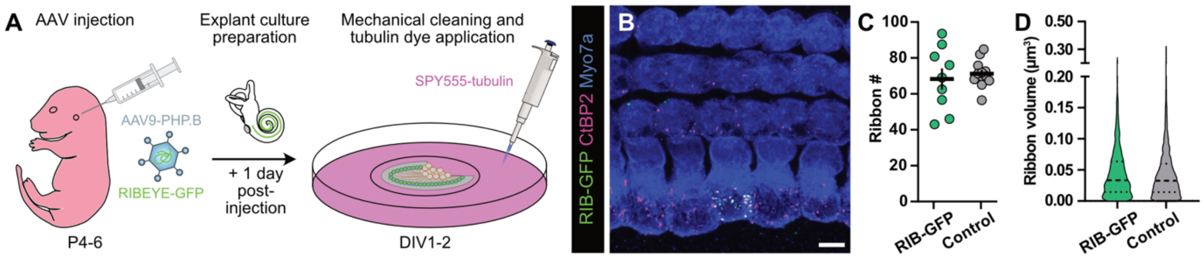

Supplemental Figure 2-S1: **Experimental paradigm and effects of short-term RIBEYE-GFP overexpression on ribbon count and volumes.**

A Wild-type mouse pups were injected with an AAV encoding RIBEYE-GFP at postnatal day P4-6. One day after transduction, organ of Corti explant cultures were prepared and – after additional one to two days *in vitro* (DIV) – mechanically-cleaned and incubated with the MT dye SPY555-tubulin. **B** Representative maximum projection of a confocal z-stack showing a transduced IHC, which expresses RIBEYE-GFP (green). Please note the colocalization with the ribbon marker CtBP2 (magenta). **C-D** Both, ribbon counts (C) and volumes (D) were indistinguishable between RIBEYE-GFP transduced and neighboring non-transduced IHCs, suggesting appropriate integration of the fluorescent construct into endogenous ribbons while not displaying any obvious overexpression artifacts. No statistical significances detected (Mann-Whitney U test). RIBEYE-GFP transduced: N=9, n=9; Control non-transduced: N=12, n=14. Scale bar: 5 µm.

bioRxiv preprint doi: https://doi.org/10.1101/2024.04.12.589153; this version posted April 16, 2024. The copyright holder for this preprint (which was not certified by peer review) is the author/funder, who has granted bioRxiv a license to display the preprint in perpetuity. It is made available under aCC-BY 4.0 International license.

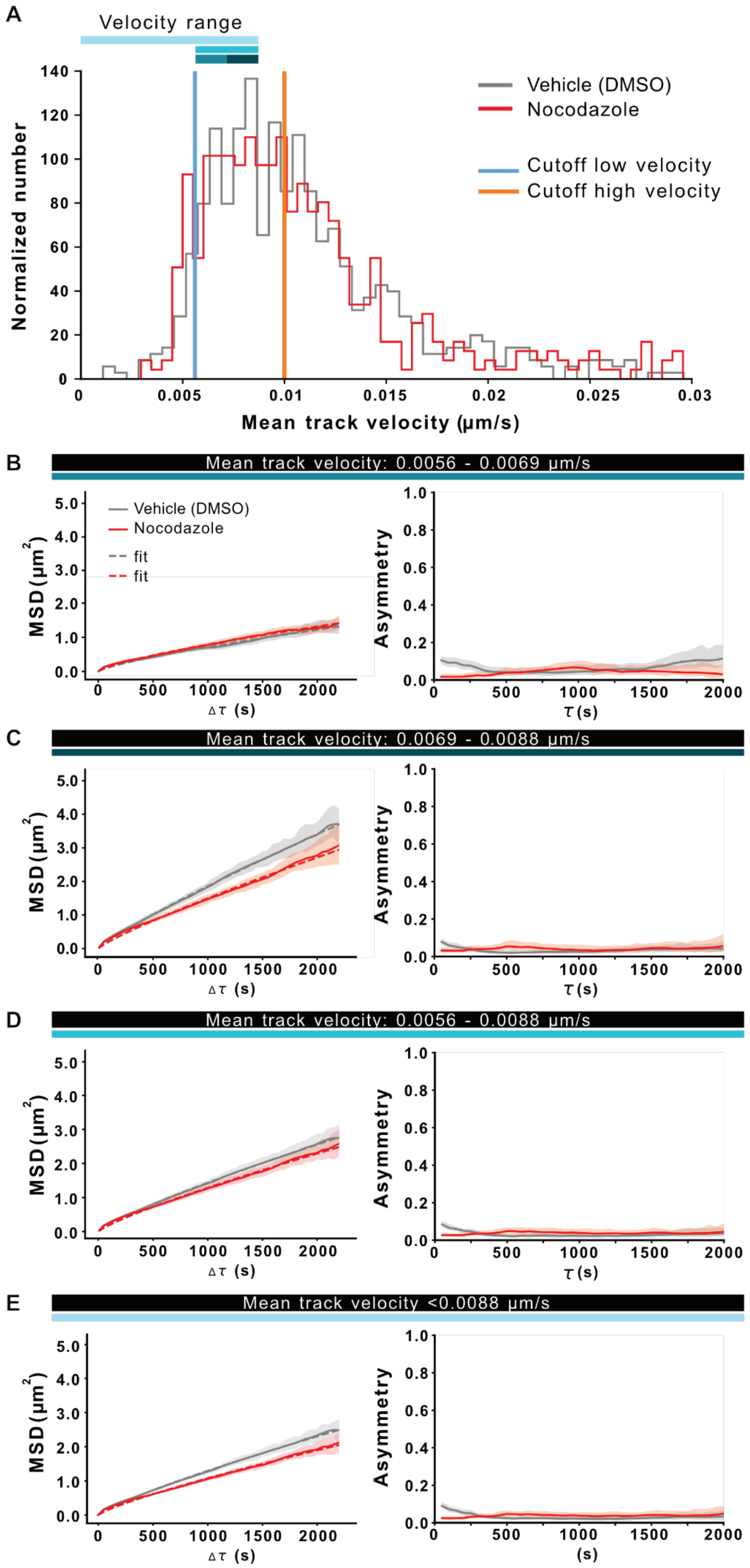

914

bioRxiv preprint doi: https://doi.org/10.1101/2024.04.12.589153; this version posted April 16, 2024. The copyright holder for this preprint (which was not certified by peer review) is the author/funder, who has granted bioRxiv a license to display the preprint in perpetuity. It is made available under aCC-BY 4.0 International license.

Supplemental Figure 5-S1: Dilution of nocodazole effects in faster-displacing ribbon precursor populations

A Reproduction of the same dataset as in **Figure 5C**: Shown is the distribution of the mean track velocity for precursors in vehicle- and nocodazole-treated IHCs. Indicated are the used cutoffs to selectively analyze trajectories with a low (blue) and high velocity (orange) displacement. Color-coded bars indicate the different velocity ranges displayed in B-E. **B-E** MSD analysis (left panels) and asymmetry assessment (right panels) of trajectories with a mean velocity within the range 0.0056 – 0.0069 µm/s (B), 0.0069 – 0.0088 µm/s (C), 0.0056 – 0.0088 µm/s (D), and below 0.0088 µm/s (E). The inclusion of a moderate-to-fast displacing population of ribbon precursor trajectories dilutes the reducing effect of nocodazole on ribbon precursor displacement and directionality present in the low velocity trajectories (below 0.0056 µm/s, as seen in **Figure 5D**). **B-D** Trajectories with a velocity between 0.0056 and 0.0088 µm/s do not appear to be subjected to directed transport in control, nor nocodazole-treated conditions. **E** Addition of the 0.0056 – 0.0088 µm/s velocity range to the low velocity cutoff range (<0.0056 µm/s) largely eliminates the distinction in 3D displacement and directed transport of ribbon precursors between nocodazole-treated and control IHCs.
